# Oxaliplatin- versus cisplatin-based regimens for elderly individuals with advanced gastric cancer: a retrospective cohort study

**DOI:** 10.1186/s12885-022-09581-6

**Published:** 2022-04-26

**Authors:** Takashi Chinen, Yusuke Sasabuchi, Hiroki Matsui, Hironori Yamaguchi, Hideo Yasunaga

**Affiliations:** 1 Department of Clinical Oncology, Jichi Medical University, 3311-1 Yakushiji, Shimotsuke-Shi, Tochigi 329-0498 Japan; 2 Data Science Center, Jichi Medical University, Tochigi, Japan; 3 Department of Clinical Epidemiology and Health Economics, School of Public Health, University of Tokyo, Tokyo, Japan

**Keywords:** Frail elderly, Stomach neoplasms, Antineoplastic agents, Chemotherapy-induced febrile neutropenia, Administrative claims, healthcare

## Abstract

**Background:**

Whether an oxaliplatin- or cisplatin-based regimen is more optimal for treating elderly patients with advanced gastric cancer, in terms of survival and adverse events remains unclear.

**Methods:**

In this retrospective cohort study, we used stacked claim data of residents in two Japanese prefectures collected between 2012 and 2017 and between 2014 and 2019, respectively. We included patients with advanced gastric cancer who received oxaliplatin-based and cisplatin-based regimens. Propensity score overlap weighting analysis was conducted to compare overall survival and granulocyte colony-stimulating factor use during chemotherapy between the oxaliplatin- and cisplatin-based treatment groups.

**Results:**

A total of 242 patients were included in the study. After propensity score weighting, Kaplan–Meier analysis showed no significant differences in overall survival between the two groups (hazard ratio: 1.13; 95% confidence interval, 0.60–2.11; *p* =  0.70). However, the proportion of patients receiving granulocyte colony-stimulating factor was significantly lower in the oxaliplatin group than in the cisplatin group (2.3% vs.22.7%, *p* = 0.01).

**Conclusions:**

Survival did not differ significantly between elderly patients with advanced gastric cancer treated with oxaliplatin-based versus cisplatin-based regimens; however, the oxaliplatin-based regimen was associated with less granulocyte colony-stimulating factor use.

**Supplementary Information:**

The online version contains supplementary material available at 10.1186/s12885-022-09581-6.

## Background

Several guidelines recommend a platinum-fluoropyrimidine doublet regimen (i.e., oxaliplatin or cisplatin combined with S-1 or capecitabine) as first-line chemotherapy for patients with advanced gastric cancer based on the results of randomised controlled trials [1–3]. Although cisplatin combined with S-1 became the standard chemotherapy regimen, adverse events, including myelotoxicity, have been reported; this has often led to discontinuation or delay of the chemotherapy schedule [4]. The G-SOX and the REAL2 trials revealed that patients administered with oxaliplatin- and cisplatin-based regimens had similar overall survival rates, although the former showed less toxicity than the latter [5, 6]. Additionally, subgroup analysis of the G-SOX trial data showed that oxaliplatin plus S-1 was non-inferior to cisplatin plus S-1 for treating patients with advanced gastric cancer who were 70 years of age or older [7].

However, elderly patients with frailty or multimorbidity who are vulnerable to the adverse effects of chemotherapy are often excluded from randomised controlled trials because of restrictive eligibility criteria [8–11]. The proportions of patients aged 70 years or older were 17% in the SPIRITS trial [4] and 31% in the G-SOX trial [5], which were much lower than the overall proportion of elderly patients with gastric cancer in Japan [12]. Therefore, data derived from elderly patients in randomised controlled trials may not reflect the interventions administered to those treated in a real-world clinical setting [13–16]. Several studies reported that aging, metastases, and comorbidities increased the risk of chemotherapy-induced febrile neutropenia [17–19].

On the other hand, several studies of advanced gastric cancer have focused on elderly patients. A retrospective study of individuals with advanced gastric cancer who were 70 years or older showed no overall survival benefit from adding cisplatin to S-1 [20]. However, a recent phase III trial showed that adding oxaliplatin to capecitabine improved survival among elderly patients with advanced gastric cancer when compared with capecitabine monotherapy [21]. Another phase II trial comparing oxaliplatin plus S-1 to S-1 alone for treating patients with advanced gastric cancer who are 70 years or older is ongoing [22].

However, the effectiveness and safety of oxaliplatin-based and cisplatin-based regimens for elderly patients with advanced gastric cancer have not been adequately compared. Therefore, we compared overall survival and adverse events in patients aged 70 years or older who were administered either oxaliplatin-based or cisplatin-based regimens to treat advanced gastric cancer using real-world data.

## Methods

### Study design and data sources

We performed a retrospective cohort study using data extracted from national healthcare insurance system databases. There are three types of healthcare insurance systems in Japan: the employee-based Social Health Insurance, in which employers provide insurance coverage to employees aged < 75 years and their families; the National Health Insurance, in which municipalities provide insurance coverage to non-employees aged < 75 years and their families; and the Late Elder’s Health Insurance, in which municipalities provide insurance coverage to elderly persons aged ≥75 years [23]. In this study, we used stacked claim data from both the National Health Insurance and Late Elder’s Health Insurance databases from Kumamoto and Tochigi prefectures in Japan. In addition, the Kokuho Database (KDB), which includes vital status on the day of withdrawal from national health insurance [24], was linked to the medical claim data for Tochigi prefecture.

These databases included the following information on medical and pharmacy claims information: anonymised identification numbers, sex, birth month, date-stamped diagnoses, medications, medical procedures, and date of death [25]. The databases contained approximately 1,700,000 enrolees and represented approximately 45% of the residents of the two prefectures. The database contained data collected between April 2012 and February 2017 for Kumamoto prefecture and between June 2014 and February 2019 for Tochigi prefecture.

### Study population

The inclusion criteria were 1) a diagnosis of gastric or esophagogastric junction cancer (International Classification of Diseases, Tenth Revision codes of C16.0–C16.9 and C15.8) during the observation period [26] and 2) being prescribed both oral fluoropyrimidine (i.e., S-1 or capecitabine) and platinum (i.e., oxaliplatin or cisplatin) [1–3]. Patients were ineligible if 1) their insurance claims included diagnostic tests and antineoplastic agents related to colorectal or lung cancer given that oxaliplatin with capecitabine and cisplatin with S-1 are administered to patients with these cancers [27, 28], 2) they had not received oral fluoropyrimidine between 11 days before and 1 day after the date of receiving platinum, 3) they had been administered chemotherapy agents other than platinum with fluoropyrimidine as their initial chemotherapy for advanced gastric cancer, 4) they had not been observed for at least 6 months before the initiation of chemotherapy, 5) they had started oxaliplatin within 60 days after gastrectomy or colectomy (thereby excluding patients receiving postoperative adjuvant chemotherapy), 6) they had received trastuzumab (hence excluding patients with human epidermal growth factor receptor 2 [HER2] positive advanced gastric cancer), and 7) they were under 70 years of age.

### Exposures and outcomes

The goal of the study was to compare ‘oxaliplatin plus S-1 or capecitabine’ to ‘cisplatin plus S-1 or capecitabine’. Initiation of chemotherapy for the oxaliplatin group was defined as the date of initial dose of oxaliplatin, whereas initiation of cisplatin with capecitabine was deemed the date of the initial cisplatin dose. Initiation of cisplatin plus S-1 was defined as 7 days before the date of the first cisplatin dose because majority of patients in Japan receive cisplatin 8 days after the initiation of S-1 based on the SPIRITS trial [4, 29].

The date of death was extracted from the data from insurance claims from Kumamoto prefecture. If the claims had no information about death, follow-up was censored on the day of the latest claim. On the other hand, the date of death was extracted from the KDB system for data from Tochigi prefecture.

The primary outcome measure of this study was overall survival, while the secondary outcome was the utilisation of granulocyte colony-stimulating factor (G-CSF) administration between the initiation of chemotherapy to 28 days after the last administration of oxaliplatin or cisplatin.

### Other variables

Other investigated covariates included age, sex, a dummy variable of the regions, comorbidities, and medications. Comorbidities were determined based on the components of the Charlson comorbidity index within the 6 months prior to the initiation of chemotherapy using algorithms developed by Quan et al. [30]. Medications administered within the 6 months prior to the initiation of chemotherapy were extracted based on the Anatomical Therapeutic Chemical classification [31].

### Statistical analysis

All descriptive statistics are reported as counts and proportions for categorical variables and as means and standard deviations or medians and interquartile ranges for continuous variables. The characteristics of the patients in the two treatment groups by two prefectures were compared.

To reduce the effect of confounding factors according to indication, we conducted propensity score weighting analyses using overlap weights [32, 33]. Propensity scores for receiving an oxaliplatin-based regimen were calculated using a multivariable logistic regression model incorporating age, sex, region, the 10 categories of the Anatomical Therapeutic Chemical classification except for the ‘antineoplastic and immunomodulating agents’ category, and the 16 Charlson comorbidity index categories other than ‘any malignancy including lymphoma and leukaemia except malignant neoplasm of skin’ because all patients had advanced gastric cancer and received antineoplastic agents. When calculating propensity scores, certain variables were excluded when the model did not converge. After estimating propensity scores, we developed overlap weights, which were proportional to 1 minus the propensity score for the oxaliplatin group and the actual propensity score for the cisplatin group. Overlap weighting created a pseudo-population that included all the analysed participants by down-weighting at both ends of the propensity score distribution as many overlaps in covariates between the two groups [33]. We calculated the mean standardised differences to assess the balance of baseline characteristics between the oxaliplatin and the cisplatin groups before and after propensity score overlap weighting. A standardized mean difference of less than 0.1 was considered as negligible imbalance between the two groups [34].

The Kaplan–Meier curves were estimated before and after propensity score weighting. The median survival times were calculated based on the Kaplan–Meier method. Overall survival between the two treatment groups was then compared in this propensity score-weighted cohort. The survival curves were constructed using the weighted Kaplan–Meier method and compared via weighted log-rank tests. To examine the effect of treatment on overall survival, hazard ratios and 95% confidence intervals were calculated using a weighted Cox proportional hazards model.

For the secondary outcome, the use of G-CSF administration was evaluated before and after propensity score weighting. After propensity score weighting, Fisher’s exact test was conducted to compare the use of G-CSF between the two groups and the odds ratios, and 95% confidence intervals were calculated.

The data of this study spans two different regions during different periods. The regions may differ in medical environments, which might introduce bias into the results. Therefore, the region of the patients in the oxaliplatin and cisplatin groups was noted before and after propensity-score weighting. In addition, the difference in time periods may introduce bias into the results. Oxaliplatin was approved on 5th September 2014 in Japan [35]. Newer drugs such as ramucirumab and nivolumab were approved for patients with gastric cancer in Japan from 22nd June 2015 and 22nd September 2017, respectively [36, 37]. However, the data used for this study are between April 2012 and February 2017 for the Kumamoto prefecture and between June 2014 and February 2019 for the Tochigi prefecture. Therefore, we conducted the following three analyses. First, the utilisation of ramucirumab and nivolumab was evaluated in different regions before propensity-score weighting. Second, utilisation of ramucirumab or nivolumab before and after propensity-score weighting was evaluated by the two treatment groups. Third, to increase sensitivity, we repeated the same analysis for primary outcomes using only the cases from the period after oxaliplatin was approved in Japan.

All statistical analyses were performed using R version 3.5.3 software (the R Foundation, Vienna, Austria). Two-sided *p* values < 0.05 were considered statistically significant.

## Results

**Fig. 1:**
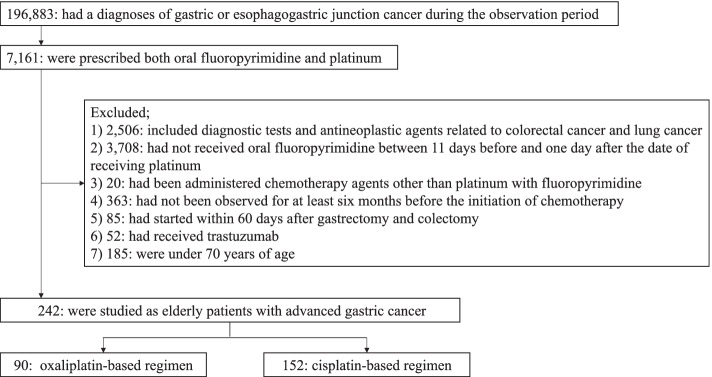
Flow diagram of the study selection process

After applying the eligibility criteria, 242 elderly patients who received platinum chemotherapy with fluoropyrimidine for advanced gastric cancer were included; the number of patients in the oxaliplatin-based and cisplatin-based treatment groups were 90 and 152, respectively (Fig. 1). The total proportion of censored cases was 41.3% (100 out of 242), and the proportion of censored cases in the oxaliplatin-based regimen of the Kumamoto prefecture was 68.6% (Table 1).

**Table 1:** Characteristics of patients in the oxaliplatin and cisplatin groups before propensity-score weighting

Characteristics	Kumamoto prefecture data		Tochigi prefecture data	
	Oxaliplatin	Cisplatin	Oxaliplatin	Cisplatin
n (%)	70 (39.8)	106 (60.2)	20 (30.3)	46 (69.7)
Mean age (standard deviation)	77.4 (4.06)	76.4 (4.23)	72.8 (1.83)	73.0 (2.01)
Sex = Female (%)	27 (38.6)	27 (25.5)	5 (25.0)	7 (15.2)
Censored cases (%)	48 (68.6)	33 (31.1)	3 (15.0)	16 (34.8)

The median follow-up time before propensity score weighting was 7.3 months (interquartile range, 3.5–10.9 months) and 9.3 months (interquartile range, 5.4–16.9 months) in the oxaliplatin-based and cisplatin-based treatment groups, respectively.

The baseline characteristics before and after propensity score overlap weighting are shown in Table 2; the baseline characteristics of the two groups were well balanced after weighting.

**Table 2:** Characteristics of patients in the oxaliplatin and cisplatin groups before and after propensity-score weighting

Characteristic	Before weighting			After weighting		
	Oxaliplatin	Cisplatin	SMD	Oxaliplatin	Cisplatin	SMD
**n**	90	152		35.3	35.3	
**Mean age (standard deviation)**	76.4 (4.16)	75.4 (4.02)	0.24	76.0 (4.19)	76.0 (4.15)	< 0.001
**Sex = Female (%)**	32 (35.6)	34 (22.4)	0.29	10.7 (30.5)	10.7 (30.5)	< 0.001
**Region = Tochigi prefecture (%)**	20 (22.2)	46 (30.3)	0.18	10.8 (30.6)	10.8 (30.6)	< 0.001
**ATC classification system**						
**Alimentary tract and metabolism (%)**	90 (100.0)	150 (98.7)	0.16	35.3 (100.0)	35.3 (100.0)	< 0.001
**Blood and blood-forming organs (%)**	90 (100.0)	151 (99.3)	0.12	35.3 (100.0)	35.3 (100.0)	< 0.001
**Cardiovascular system (%)**	74 (82.2)	116 (76.3)	0.15	30.4 (86.1)	30.4 (86.1)	< 0.001
**Dermatological (%)**	33 (36.7)	51 (33.6)	0.07	13.3 (37.8)	13.3 (37.8)	< 0.001
**Genitourinary system and sex hormones (%)**	15 (16.7)	17 (11.2)	0.16	5.1 (14.5)	5.1 (14.5)	< 0.001
**Systemic hormonal preparations, excluding sex hormones and insulin (%)**	90 (100.0)	82 (53.9)	1.31	35.3 (100.0)	35.3 (100.0)	< 0.001
**Anti-infective (systemic use) (%)**	53 (58.9)	94 (61.8)	0.06	21.9 (62.1)	21.9 (62.1)	< 0.001
**Musculo-skeletal system (%)**	64 (71.1)	113 (74.3)	0.07	26.4 (74.8)	26.4 (74.8)	< 0.001
**Nervous system (%)**	88 (97.8)	146 (96.1)	0.10	34.2 (97.1)	34.2 (97.1)	< 0.001
**Antiparasitic products, insecticides, and repellents (%)**	78 (86.7)	125 (82.2)	0.12	30.3 (85.8)	30.3 (85.8)	< 0.001
**Respiratory system (%)**	47 (52.2)	59 (38.8)	0.28	18.4 (52.3)	18.4 (52.3)	< 0.001
**Sensory organs (%)**	31 (34.4)	40 (26.3)	0.18	11.0 (31.2)	11.0 (31.2)	< 0.001
**Various (%)**^a^	90 (100.0)	148 (97.4)	0.23	35.3 (100.0)	34.6 (98.0)	0.20
**Comorbidities**^b^						
**Myocardial infarction (%)**	3 (3.3)	12 (7.9)	0.20	1.5 (4.2)	1.5 (4.2)	< 0.001
**Congestive heart failure (%)**	18 (20.0)	35 (23.0)	0.07	6.7 (19.1)	6.7 (19.1)	< 0.001
**Peripheral vascular disease (%)**	8 (8.9)	21 (13.8)	0.16	4.4 (12.5)	4.4 (12.5)	< 0.001
**Cerebrovascular disease (%)**	11 (12.2)	21 (13.8)	0.05	4.9 (14.0)	4.9 (14.0)	< 0.001
**Dementia (%)**	1 (1.1)	0 (0.0)	0.15	0.0 (0.0)	0.0 (0.0)	< 0.001
**Chronic pulmonary disease (%)**	28 (31.1)	34 (22.4)	0.20	10.4 (29.5)	10.4 (29.5)	< 0.001
**Rheumatic disease (%)**	1 (1.1)	7 (4.6)	0.21	0.6 (1.8)	0.6 (1.8)	< 0.001
**Peptic ulcer disease (%)**	26 (28.9)	52 (34.2)	0.14	12.1 (34.2)	12.1 (34.2)	< 0.001
**Mild liver disease (%)**	26 (28.9)	54 (35.5)	0.14	10.6 (30.1)	10.6 (30.1)	< 0.001
**Diabetes without chronic complication (%)**	14 (15.6)	20 (17.1)	0.04	5.7 (16.2)	5.7 (16.2)	< 0.001
**Diabetes with chronic complication (%)**	1 (1.1)	8 (5.3)	0.24	0.7 (1.9)	0.7 (1.9)	< 0.001
**Hemiplegia or paraplegia (%)**	1 (1.1)	2 (1.3)	0.02	0.3 (0.8)	0.3 (0.8)	< 0.001
**Renal disease (%)**	4 (4.4)	3 (2.0)	0.14	0.8 (2.2)	0.8 (2.2)	< 0.001
**Moderate or severe liver disease (%)**	1 (1.1)	0 (0.0)	0.15	0.0 (0.0)	0.0 (0.0)	< 0.001
**Metastatic solid tumor (%)**	65 (72.2)	120 (78.9)	0.16	27.6 (78.2)	27.6 (78.2)	< 0.001
**AIDS/HIV (%)**	90 (100.0)	152 (100.0)	< 0.001	35.3 (100.0)	35.3 (100.0)	< 0.001

*SMD* standardized mean difference, *ATC* anatomical therapeutic chemical, *AIDS* acquired immunodeficiency syndrome, *HIV* human immunodeficiency virus. ^a^ This category was removed when calculating the propensity score. ^b^Comorbidities were according to the Charlson comorbidity index

**Fig. 2:**
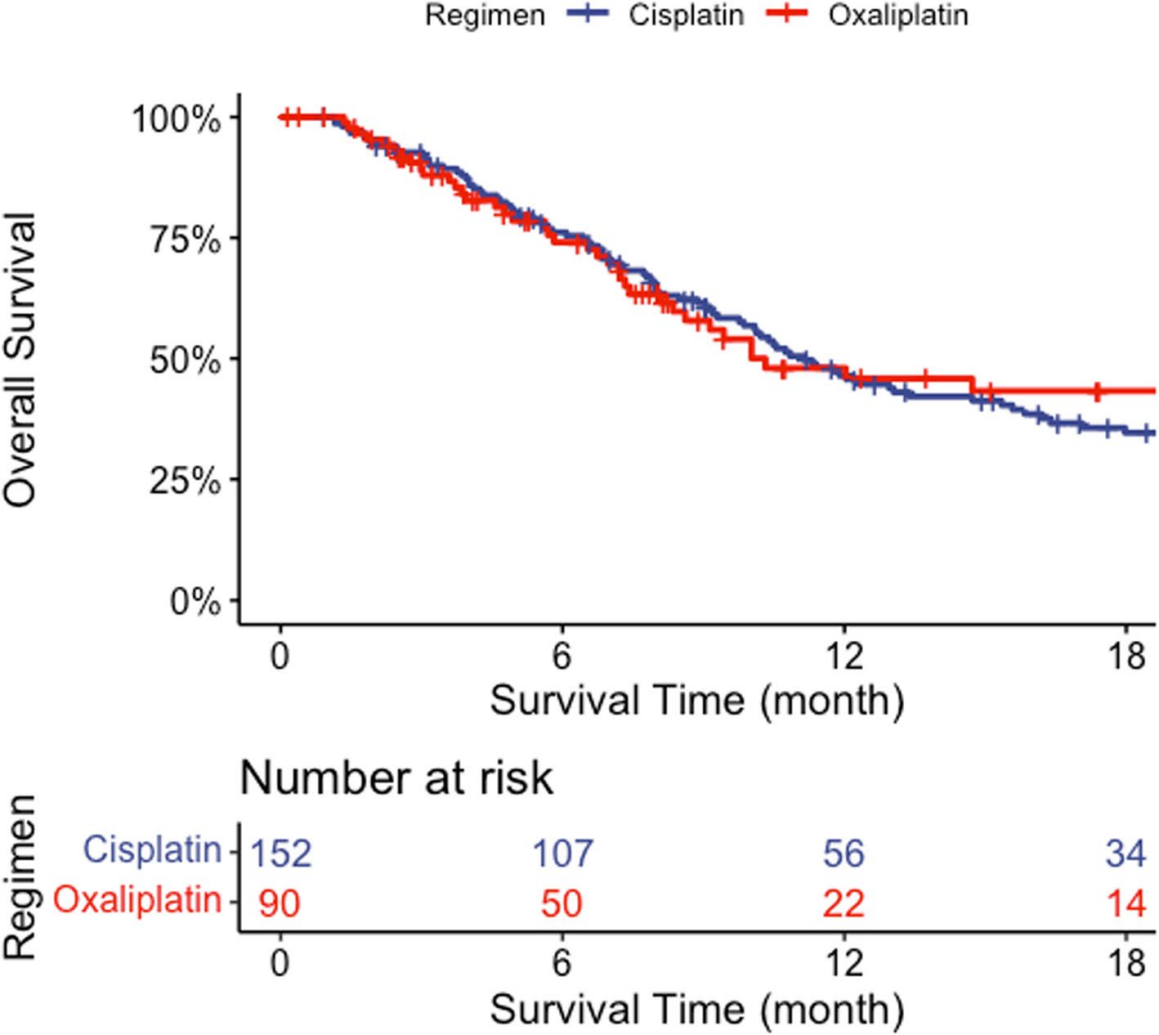
Kaplan–Meier survival curves for the two groups before propensity-score weighting. These unweighted Kaplan–Meier curves showed the overall survival of elderly patients with advanced gastric cancer treated with either the oxaliplatin-based or the cisplatin-based regimens. The median overall survival before propensity score weighting was 10.5 months (95% confidence interval, 8.5 - Not Available months) in the oxaliplatin-based and 11.3 months (95% confidence interval, 9.9–15.6 months) in the cisplatin-based treatment groups

The Kaplan–Meier curve before propensity score weighting is shown in Fig. 2.

**Fig. 3:**
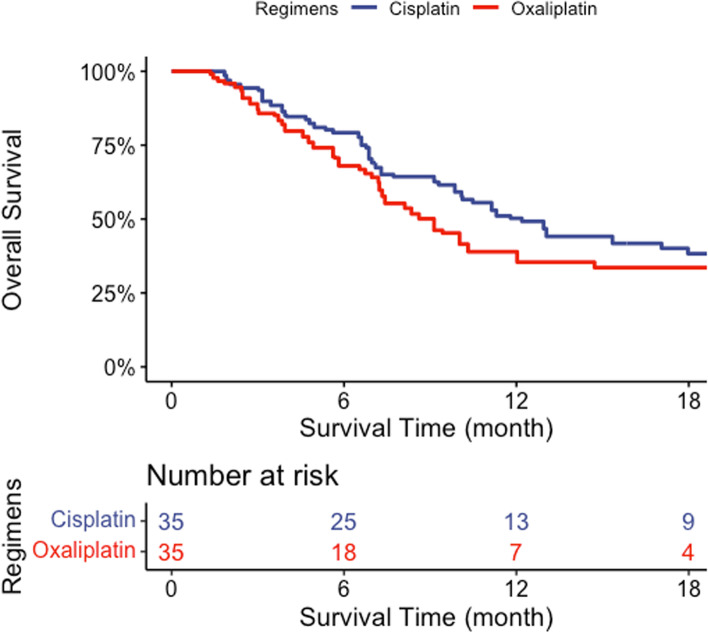
Propensity score-weighted Kaplan–Meier survival curves for the two groups. These weighted Kaplan–Meier curves showed the overall survival of elderly patients with advanced gastric cancer treated with either oxaliplatin-based or cisplatin-based regimens. The median overall survival was 9.3 months (95% confidence interval, 7.1- Not Available months) and 12.4 months (95% confidence interval, 7.8–26.8 months) in the oxaliplatin-based and cisplatin-based treatment groups, respectively. The weighted log-rank test showed a *p*-value of 0.58. The hazard ratio was 1.13 (95% confidential interval, 0.60–2.11; *p* = 0.70)

According to our Kaplan–Meier analysis, no significant differences in survival were observed between the two groups (weighted log-rank test *p* = 0.58) (Fig. 3). The hazard ratio was 1.13 (95% confidence interval, 0.60–2.11; *p* = 0.70).

Before propensity score weighting, 2/90 (2.2%) and 36/152 (23.7%) of the patients received G-CSF during chemotherapy in the oxaliplatin- and the cisplatin-based treatment groups, respectively. After propensity score weighting, the proportion of patients who received G-CSF in the oxaliplatin-based regimen group was significantly lower than that in the cisplatin-based regimen group (2.3% vs. 22.7%, *p* = 0.01).

The proportion of patients on ramucirumab and nivolumab is shown in Supplemental Table 1. Ramucirumab and nivolumab were more frequently used in the Tochigi prefecture; however, the proportion of patients with oxaliplatin-based regimens in the Tochigi prefecture was 30%, and that in the Kumamoto prefecture was 40% (Table 1). Supplemental Table 2 shows the drug utilisation of patients in the oxaliplatin and cisplatin groups before and after propensity-score weighting. After propensity-score weighting, the proportions of ramucirumab use were higher in the oxaliplatin group than in the cisplatin group. A sensitivity analysis showed results similar to the main analysis (hazard ratio: 1.52, 95% confidence interval: 0.77–2.99).

## Discussion

Our study was the first to investigate the risks and benefits of the platinum-fluoropyrimidine doublet regimens for elderly individuals with advanced gastric cancer in a real-world setting. We found that the overall survival was not significantly different between elderly patients with advanced gastric cancer treated with fluoropyrimidine combined with oxaliplatin versus fluoropyrimidine combined with cisplatin in a real-world clinical setting. On the other hand, the frequency of administration of G-CSF was significantly less in patients who received the oxaliplatin with fluoropyrimidine than in those who received the cisplatin with fluoropyrimidine.

A previous study by Makiyama et al. showed no benefit of combining cisplatin with S-1 [20]; however, another study by Hwang et al. demonstrated a survival benefit from combining oxaliplatin to capecitabine [21]. However, the data from our present study were not consistent with previously obtained results. The reason for this discrepancy is unclear, although the reduced dosing of platinum agents may influence outcomes and may have resulted in extending overall survival because of less toxicity to elderly patients. An initial dose of oxaliplatin plus capecitabine of 130 mg/m^2^ is accepted as a standard regimen based on the REAL2 trial [6]. Only 27.5% of patients in Makiyama et al.’s study received a reduced initial dose of cisplatin [20], whereas all patients in Hwang et al.’s study (which was set in Korea) were administered a reduced initial dose of oxaliplatin plus capecitabine (110 mg/m^2^) [23]. In a real-world clinical setting, most elderly patients are administered reduced doses of palliative chemotherapy [38, 39], and in Japan, the standard regimen of oxaliplatin plus S-1 is an initial dose of 100 mg/m^2^ for patients with advanced gastric cancer based on the G-SOX trial [5, 40].

The proportion of patients with febrile neutropenia in the cisplatin group was approximately 3% in a previous study [4], whereas the incidence of grade 3 or 4 neutropenia was reported to be 20.2% [20]. In the present study, more than 20% of the patients in the cisplatin group received G-CSF. It is suggested that G-CSF was used for patients with neutropenia without fever as secondary prophylaxis in Japan. This inappropriate overuse may have been done because of the multiple risk factors of the patients with neutropenia for febrile neutropenia [41]. A previous study showed that maintaining the dosage intensity of the chemotherapeutic drugs along with prophylactic use of G-CSF had a positive effect on survival [42], although this is not recommended by clinical practice guidelines [17, 18]. The use of oxaliplatin may reduce the inappropriate use of G-CSF for patients with advanced gastric cancer.

Based on the results of this study as well as the fact that oxaliplatin can be administered to outpatients [43], an oxaliplatin-based regimen may be preferable to a cisplatin-based counterpart for treating elderly patients with advanced cancer.

Our study had some limitations. First, we used two different claim databases; the accuracies of the diagnoses therein were not validated and information on cancer stage was not included. However, we considered patients with a diagnosis of either gastric cancer or esophagogastric junction cancer who were prescribed anti-gastric cancer agents to have advanced gastric cancer. Second, short observation periods may be the reason for the high proportion of censored cases. Oxaliplatin was covered by insurance the treatment of advanced gastric cancer in September 2014 in Japan and the database of the Kumamoto prefecture contains data from April 2012 until February 2017. Third, the regions studied may differ in medical environments, which may result in a potential bias in the results. However, propensity-score weighting successfully balanced the regions between the two treatment groups. The propensity-score weighting may mitigate bias due to differences in regional medical environments. Fourth, the different treatment periods may cause bias in the results; therefore, the following three analyses were conducted. (1) Frequent ramucirumab or nivolumab utilisation in the Tochigi prefecture before propensity-score weighting may cause bias in the results; however, the proportion of patients on oxaliplatin-based regimens in the Tochigi prefecture was lower than that in the Kumamoto prefecture. The use of two datasets may not have a significant impact on comparing the two treatment groups. (2) The reason for frequent utilisation of ramucirumab in the oxaliplatin group may be because patients in the oxaliplatin group were frailer compared with the patients in the cisplatin group; such patients are often unable to continue the first-line regimen, and this may be associated with a high rate of transition to the second-line regimen. However, the impact of frequent use of ramucirumab on the results may be limited. Subgroup analysis of the RAINBOW trial showed that Japanese patients had no overall survival benefit [44]. (3) Although the sample size was small, the results of the sensitivity analysis did not show a significant difference between the two groups. Further, since the database did not include information on performance status, our results may have been skewed by the selection of regimens according to the patients’ conditions. Elderly individuals with poor performance status tend to receive oxaliplatin, which is believed to be associated with fewer adverse effects than cisplatin [5]. Therefore, the lack of data on performance status, and our inability to adjust for it, may have biased the data toward oxaliplatin being associated with worse survival. In addition, the sample size of the present study may not have been sufficient to determine survival differences between the groups. Lastly, the databases lacked geriatric assessments [45], which would have led to a reduction in the antineoplastic agent doses [46, 47]. The databases also did not include information on body surface area vis-à-vis the dosages of the antineoplastic agents.

## Conclusion

The oxaliplatin-based regimen may have comparable survival benefits with the cisplatin-based regimen among elderly patients with advanced gastric cancer in a real-world clinical setting. Further, the frequency of administration of G-CSF was significantly less in patients who received the oxaliplatin-based regimen.

## Supplementary Information

**Additional file 1: Supplemental Table 1.** Drug utilization of patients from Kumamoto and Tochigi prefecture data before propensity score weighting.

**Additional file 2: Supplemental Table 2.** Drug utilisation of patients before and after propensity-score weighting.

## Data Availability

Data cannot be made publicly available for ethical reasons as the data are patient data. The data are available to interested researchers upon request to Yusuke Sasabuchi (e-mail: sasabuchi@jichi.ac.jp), pending ethical approval.

## Abbreviations

G-CSF: Granulocyte colony-stimulating factor
HER2: Human epidermal growth factor receptor 2
KDB: Kokuho DataBase
