# Supplementary material for: Oxaliplatin- versus cisplatin-based regimens for elderly individuals with advanced gastric cancer: a retrospective cohort study
Source: BMC Cancer. 2022 Apr 26;22:460. doi: 10.1186/s12885-022-09581-6 (PMC9044765; doi:10.1186/s12885-022-09581-6)
Supplement: Supplementary file 1 — Additional file 1: Supplemental Table 1. Drug utilization of patients from Kumamoto and Tochigi prefecture data before propensity score weighting. [file 12885_2022_9581_MOESM1_ESM.docx]

Supplemental Table 1. Drug utilization of patients from Kumamoto and Tochigi prefecture data before propensity score weighting.

|  | **Kumamoto prefecture data** | | **Tochigi prefecture data** | |
| --- | --- | --- | --- | --- |
| Drug type | **Oxaliplatin** | **Cisplatin** | **Oxaliplatin** | **Cisplatin** |
| n | 70 | 106 | 20 | 46 |
| Ramucirumab (%) | 12 (17.1) | 12 (11.3) | 6 (30) | 13(28.3) |
| Nivolumab (%) | 0 (0.0) | 0 (0.0) | 2 (10) | 3 (6.5) |
