# Supplementary material for: Oxaliplatin- versus cisplatin-based regimens for elderly individuals with advanced gastric cancer: a retrospective cohort study
Source: BMC Cancer. 2022 Apr 26;22:460. doi: 10.1186/s12885-022-09581-6 (PMC9044765; doi:10.1186/s12885-022-09581-6)
Supplement: Supplementary file 2 — Additional file 2: Supplemental Table 2. Drug utilisation of patients before and after propensity-score weighting. [file 12885_2022_9581_MOESM2_ESM.docx]

Supplemental Table 2. Drug utilisation of patients before and after propensity-score weighting.

|  | **Before weighting** | | **After weighting** | |
| --- | --- | --- | --- | --- |
| **Drug utilisation** | **Oxaliplatin** | **Cisplatin** | **Oxaliplatin** | **Cisplatin** |
| **n** | 90 | 152 | 35.3 | 35.3 |
| **Ramucirumab (%)** | 18 (20.0) | 25 (16.4) | 7.6 (21.6) | 4.0 (11.5) |
| **Nivolumab (%)** | 2 (2.2) | 3 (2.0) | 1.2 (3.5) | 1.0 (3.0) |
